# The Use of Communication Apps by Medical Staff in the Australian Health Care System: Survey Study on Prevalence and Use

**DOI:** 10.2196/medinform.9526

**Published:** 2018-02-09

**Authors:** Amanda Nikolic, Nilmini Wickramasinghe, Damian Claydon-Platt, Vikram Balakrishnan, Philip Smart

**Affiliations:** ^1^ General Surgery and Gastroenterology Clinical Institute Epworth Healthcare Richmond Australia; ^2^ Health Information Management Epworth Healthcare Richmond Australia; ^3^ Information Technology Department Epworth Healthcare Richmond Australia; ^4^ Department of Surgery Eastern Health Box Hill Australia

**Keywords:** mobile phone, information science, communications media, privacy, interdisciplinary communication, hospital communication systems, communication

## Abstract

**Background:**

The use of communication apps on mobile phones offers an efficient, unobtrusive, and portable mode of communication for medical staff. The potential enhancements in patient care and education appear significant, with clinical details able to be shared quickly within multidisciplinary teams, supporting rapid integration of disparate information, and more efficient patient care. However, sharing patient data in this way also raises legal and ethical issues. No data is currently available demonstrating how widespread the use of these apps are, doctor’s attitudes towards them, or what guides clinician choice of app.

**Objective:**

The objective of this study was to quantify and qualify the use of communication apps among medical staff in clinical situations, their role in patient care, and knowledge and attitudes towards safety, key benefits, potential disadvantages, and policy implications.

**Methods:**

Medical staff in hospitals across Victoria (Australia) were invited to participate in an anonymous 33-question survey. The survey collected data on respondent’s demographics, their use of communication apps in clinical settings, attitudes towards communication apps, perceptions of data “safety,” and why one communication app was chosen over others.

**Results:**

Communication apps in Victorian hospitals are in widespread use from students to consultants, with WhatsApp being the primary app used. The median number of messages shared per day was 12, encompassing a range of patient information. All respondents viewed these apps positively in quickly communicating patient information in a clinical setting; however, all had concerns about the privacy implications arising from sharing patient information in this way. In total, 67% (60/90) considered patient data “moderately safe” on these apps, and 50% (46/90) were concerned the use of these apps was inconsistent with current legislation and policy. Apps were more likely to be used if they were fast, easy to use, had an easy login process, and were already in widespread use.

**Conclusions:**

Communication app use by medical personnel in Victorian hospitals is pervasive. These apps contribute to enhanced communication between medical staff, but their use raises compliance issues, most notably with Australian privacy legislation. Development of privacy-compliant apps such as MedX needs to prioritize a user-friendly interface and market the product as a privacy-compliant comparator to apps previously adapted to health care settings.

## Introduction

Due to increased availability, affordability, and functionality, the use of mobile phones to communicate and enhance clinical practice within Australian hospitals is widespread [1-3]. Thousands of apps developed by third parties are available for use on mobile phones to aid in clinical decision making, monitoring of patients, medical education or information, communication, and more [4,5].

Using mobile phones for communication is possible through multiple means including texting (short message service [SMS]), voice and video calling, conferencing email, multimedia messaging, and communication apps such as WhatsApp and Viber [4].

Effective and efficient communication is key to safe and high quality patient care. In hospitals, challenges to communication include large multidisciplinary teams with complex hierarchies guiding patient care, a proliferation of clinical information that is often time critical, and the necessity of staff travel within hospitals and between health care sites. Traditional communication platforms such as paging may be unreliable, and 2-way communication is difficult. The use of communication apps on mobile phones to communicate with colleagues is fast, efficient, portable, and convenient [6-8]. These apps are often free, easily available, and in widespread use. They facilitate rapid communication within teams through conversation and closed-group features, overview and increased involvement by senior clinicians, an enhanced patient handover process, easy communication of patient results, and rapid changes to patient management plans [5,7,8]. With multiple health care staff caring for patients, enhanced communication supports greater efficiency [8]. Therefore, communication app use offers an attractive mode of communication for health care professionals.

The advancement of technology allowing communication on portable devices brings with it a range of legal and ethical issues. In Australia, federal, state, and territory privacy laws regulate the handling of personal information. Consent must be obtained for the use of such information, which can only be used for the purpose consented to. An obligation arises upon entities handling this information to ensure compliance with prescribed privacy principles. For example, security of the information must be catered for to protect it from any unauthorized use or disclosure [7]. Most of the mobile phone apps currently in general usage within the Victorian health care system to communicate clinical information do not comply with these regulations. Consent is often not obtained, data may be accessed from the host device if it is lost or hacked, the data may be stored on an insecure server, which is often overseas or backed up overseas [9]. In Australia, certain apps have been developed to comply with Australian privacy regulations, such as MedX and MyBeepr.

A small number of studies have demonstrated the benefits of communication apps when used as an intervention in clinical practice [4-6]. However, no data exists on whether these apps are being used by medical staff. It is also unclear why medical staff use one app over another. This study aimed to quantify and qualify the use of communication apps among medical staff in clinical situations, their role in patient care, and to elaborate on issues relating to safety, key benefits, potential disadvantages, and policy implications.

## Methods

### Recruitment

Medical staff across Victorian hospitals from September to October 2017 were sent an email, social media post, or were personally approached to complete an anonymous 33-item online survey administered by SurveyMonkey (Multimedia Appendix 1). The survey was trialed on 2 medical staff at 1 hospital—their responses were not included in the results. “Logic” was used at some questions to guide respondents to further questions based on their previous response. The number of medical staff reached was unable to be calculated given the nature of social media posts and emails that were sent generally to medical staff across multiple departments. The lead researcher and 2 other researchers disseminated the survey. A brief description of the survey, details about anonymity, and intention to publish de-identified data was outlined in the first page of the survey. Consent was obtained with continued participation in the survey beyond the first page signaling consent.

### Data Collection

The survey collected data on the following: (1) respondents demographics, (2) use of communication apps in clinical practice, (3) amount and type of communication app use, (4) attitudes towards communication apps, (5) perceived benefits and disadvantages, (6) views on data “safety”, and (7) why one communication app was chosen over others.

## Results

### Demographics

In total, 118 responses were received, of which 88 (74.6%, 88/118) were complete responses. Of the respondents, 67.8% (80/118) were doctors, with 32.2% (38/118) medical students. The majority of respondents worked in the surgical field (Table 1). Most people (72.2%, 83/115) owned an iPhone.

### Communication App Use

Most participants used WhatsApp (85.0%, 96/113) as their main app for communicating clinical information. Most respondents used the app daily (78.4%, 80/102), with the median number of messages sent being 12 per day. A range of patient information was shared on communication apps, both with individual colleagues as well as within clinical teams (Table 2).

### Knowledge and Perceptions of Safety and Privacy

Most participants (67%, 60/90) thought communicating patient information on apps was only moderately safe, with 21% (19/90) considering the information safe. Of the participants, 50% (46/90) felt they may “get into trouble” by sharing patient information on apps and 76% (64/86) did not know that if communicated data was stored on overseas servers it breached Australian privacy legislation. Only 45% (5/11) of participants were aware of a hospital policy regarding the use of apps. Most participants were aware that patient consent was required to share the information. The majority of participants (94%, 85/90) considered that consent was required prior to “taking a photograph of a wound to send to a plastic’s registrar”. However, only half considered this consent needed to be documented in the patient notes or entered onto a hospital consent form.

### Perceptions on the Benefits and Disadvantages of Using Apps for Clinical Purposes

All respondents stated benefits relating to the use of apps in clinical practice. These included prompt communication, reduction in interruptions, portability, easier access to senior clinicians and other team members who were only intermittently available (ie, when registrars or consultants are scrubbed in theater), and enhanced communication relating to patient progress, results, and education. Other benefits noted included the ability to create “groups” correlating to clinical teams, being able to view who has seen comments, and the ability to mute conversations when not at work. Most respondents (78%, 71/90) also noted disadvantages relating to the use of apps. The main disadvantage noted by 94% (64/68) of participants was the potential risk to patient confidentiality. Less commonly viewed disadvantages included 1-sided communication, missing aspects of a conversation thread, and the expectation that all members of the group were equally informed about patient information shared on the app, even if members of the group were not present in the workplace. In addition, there was a concern that use of apps needed to be commensurate with the clinical situation, with face to-face or voice interaction required for more time critical situations.

### Preferences

Respondents were more likely to use an app that was free, easy to login, in wide usage with other colleagues, enabled the establishment of discrete “groups,” permitted the sharing of multiple data formats (ie, text, images, tables, video), and complied with privacy requirements. Only 13% (11/85) of participants were aware of the communication app MedX; however, none used it as their main communication app. Most frequently cited reasons for this included (1) a difficult login process and user interface; (2) widespread use of WhatsApp as an alternative; (3) and the perception that messages degrade on MedX.

The key findings of the surveys are shown in Textbox 1.

**Table 1:** Demographics of respondents.

Demographics		n (%)
**Position (N=115)**			
	Medical student	38 (32.2)
	Intern	19 (16.1)
	Resident	17 (14.4)
	Registrar	28 (23.7)
	Fellow	8 (6.8)
	Consultant	8 (6.8)
**Department (N=71)**			
	Medicine	39 (35)
	Surgery	51 (45)
	Pediatrics	1 (1)
	Obstetrics and Gynecology	2 (2)
	Radiology	3 (3)
	Emergency	9 (12)
**Phone (N=115)**			
	iPhone	83 (72.2)
	Android	32 (27.8)

**Table 2:** Communication app use, types of information shared, and group communications.

Characteristics		n (%)
**Main app used for clinical purposes (N=105)**			
	WhatsApp	89 (84.7)
	Viber	0
	MedX	0
	Slack	1 (0.9)
	Other	15 (14.3)
**Quantity/use of app for clinical purposes (N=100)**			
	Daily	78 (78)
	<Daily	22 (22)
**Type of information sent via communication app**			
	Patient management details	78 (80)
	Patient results	77 (79)
	Details that facilitate clinical handover	63 (65)
	Questions to colleagues about management	69 (79)
	Answers to colleagues about management	65 (75)
	Pictures of bradma labels	49 (56)
	Patient name and unit record numbers (unique patient identifier)	62 (71)
	Pathology results	66 (76)
	Admission notes	38 (44)
	Imaging reports	54 (61)
	Pathology reports	51 (44)
	Microbiology reports	35 (50)
	Radiological pictures	49 (56)
	Interventional reports^a^	45 (52)
	Electrocardiogram images	33 (38)
Participants belong to a communication or team group (N=96)		87 (91)

^a^Examples of interventional reports include reports and operation notes.

**Key findings of the surveys.**

- All medical staff owned a mobile phone.
- The majority of medical staff used apps for clinical purposes on a daily basis.
- WhatsApp was the most commonly-used app.
- All staff shared patient data.
- All staff considered apps to enhance clinical practice and communication to improve patient care.
- Confusion existed regarding what consent was required when sharing patient information.
- Most medical staff were concerned about the privacy implications of apps for clinical purposes.
- Staff were more likely to use an app if it was in widespread use, and was free, reliable, and easy to use.

## Discussion

### Communication App Use and Benefits

The use of apps for clinical purposes by medical staff is widespread, with most using them to enhance communication with colleagues and share clinical information to enhance patient outcomes. All participants saw the benefit of using apps in clinical situations, considering them to be efficient, portable, and a less obtrusive means of facilitating patient handover, communicating within teams, integrating patient information, and optimizing patient management plans. It is clear the role of these apps in Victorian clinical practice is well-established and offers benefits over more traditional forms of communication (ie, paging systems, voice calling, and face to face meetings). In particular, they are suited to the unique challenges faced by health care teams including large multidisciplinary teams where senior staff may only be intermittently available, responding to time critical issues, and optimizing team interaction in geographically-diverse health care centers.

### Knowledge and Perceptions of Safety and Privacy

Despite the widespread use of apps, there was confusion about privacy implications and consent. For example, most participants knew that consent was required when taking a photograph on a mobile phone to share with a colleague; however, only half considered that documenting patient consent in their notes was also required. Added complexity exists when considering ongoing discussion and sharing of a wide range of patient information to facilitate daily patient care by medical staff on communication apps. Privacy legislation in Australia states that patient information can only be used for the purpose to which it was collected and consented for by the patient [7]. Medico-legal providers recommend documenting consent in patient notes when sharing images on mobile phones [10]. This raises the question of whether patient consent should also be obtained prior to sharing their information on communication apps. Given the number of patients often admitted under clinical teams, this requirement may serve to reduce the speed, efficacy, and attractiveness of these apps. Another option may be to require consent on admission by patients for the specified and agreed purpose of discussing their care via these apps.

Respondents associated subjective risks with sharing patient data on communication apps, with half suspecting they may get “in trouble” for doing so, and 97% (64/68) acknowledging privacy concerns. However, the majority of medical staff continue to use these apps on a daily basis despite recognizing potential non-compliance with privacy laws. This may be considered a case of convenience and familiarity trumping privacy, or alternatively clinicians becoming reliant on their mobile phones and these types of communication technologies for patient-related communication. Medical staff were also unaware that these apps store data, including identifiable patient information, on overseas servers which contravenes Australian privacy legislation. Although there have not been any legal cases against medical staff regarding the use of these apps, given their non-compliance with data safety legislation, a case may be made against medical staff if data shared on these apps was compromised. The Medical Board of Australia does not currently have guidelines that address the use of communication apps. However, in 2015 they highlighted the risks related to communication with patients via electronic messaging and recommended that medical practitioners be aware of privacy legislation [11].

Simple steps may be taken by medical staff to decrease the risk to patient data safety when using mobile phones for communication. These include obtaining consent from the patient, having pin numbers on devices to prevent unauthorized access, and deleting information once it is not longer required. The Australian Medical Association (AMA) policy on the use of clinical photography on mobile phones is a good practical guide that can be used in the clinical setting [12]. Hospitals may also play a role in increasing awareness relating to privacy by establishing their own policies.

The increasing development, use, and benefits of communication apps needs to be balanced against the risk to patient safety and confidentiality. Developing apps which comply with privacy legislation, protect patient data with encryption, and are resistant to cyber crime may facilitate the use of these apps without risking patient data security. Guidelines do not currently exist which address the use of communication apps in clinical practice. These need to be developed to guide medical staff on their safe use, at a pace which mirrors their adoption by clinical staff.

### Perceptions of Disadvantages Relating to the Use of Mobile Phone Apps

Barriers and disadvantages relating to the use of apps were acknowledged by 80% (72/90) of participants with the most commonly-cited being the risk to patient confidentiality. Other disadvantages were less commonly noted, and did not appear to deter medical staff from using these apps.

### Preferences

Respondents were more likely to use an app if it was free to access, already in widespread use, had an easy and reliable interface, and was easy to use. WhatsApp was the most frequently used app. Most staff were unaware of a purpose-built communication app called MedX, which complies with Australian privacy regulations. Medical staff that were aware of MedX did not use the app due to a difficult login process, user interface, and low use pattern. The widespread use of other apps, mainly WhatsApp, will likely render the introduction of these compliant apps problematic. A simpler login process and user interface needs to be developed, local policies prioritizing these apps, appropriate advertising, and even incentives may be required to shift established usage to privacy-compliant apps. Given the intersection of app usage with federal, state, and territory laws, this issue may also be worth considering by the Victorian Boards Ministerial Advisory Committee, and perhaps even the Australian Health Ministers’ Advisory Council (AHMAC) and COAG Health Council (CHC).

### Limitations

The limitations of this study include the small sample population and simple survey framework.

### Conclusions

The use of communication apps by medical personnel in Victorian hospitals is pervasive, with WhatsApp the most commonly used. These apps play a role in optimizing communication between medical staff to deliver better health outcomes for patients. The major disadvantage arising from the use of apps is the non-compliance of apps currently in widespread usage with Australian privacy legislation. However, this does not appear to limit their use despite the majority of medical staff acknowledging risks to patient privacy. Development of privacy-compliant apps such as MedX needs to prioritize those features that currently engage user interest in non-compliant apps. A coordinated effort is also required in a regulatory and policy sense to ensure the transition to privacy-compliant apps. This is an initiative worthy of consideration by the Victorian Boards Ministerial Advisory Committee, and perhaps even the AHMAC and CHC.

## Appendix

Multimedia Appendix 1 The SurveyMonkey questionnaire used in this study.

## Abbreviations

AHMAC: Australian Health Ministers’ Advisory Council
CHC: COAG Health Council

## References

[1] Mosa A, Yoo I, Sheets L (2012). A systematic review of healthcare applications for smartphones. BMC Med Inform Decis Mak.

[2] O'Connor P, Byrne D, Butt M, Offiah G, Lydon S, Mc IK, Stewart B, Kerin MJ (2014). Interns and their smartphones: use for clinical practice. Postgrad Med J.

[3] Charani E, Castro-Sánchez E, Moore L, Holmes A (2014). Do smartphone applications in healthcare require a governance and legal framework? It depends on the application!. BMC Med.

[4] Ventola CL (2014). Mobile devices and apps for health care professionals: uses and benefits. P&T.

[5] Ozdalga E, Ozdalga A, Ahuja N (2012). The smartphone in medicine: a review of current and potential use among physicians and students. J Med Internet Res.

[6] Patel B, Johnston M, Cookson N, King D, Arora S, Darzi A (2016). Interprofessional communication of clinicians using a mobile phone app: a randomized crossover trial using simulated patients. J Med Internet Res.

[7] Johnston MJ, King D, Arora S, Behar N, Athanasiou T, Sevdalis N, Darzi A (2015). Smartphones let surgeons know WhatsApp: an analysis of communication in emergency surgical teams. Am J Surg.

[8] Khanna V, Sambandam S, Gul A, Mounasamy V (2015). “WhatsApp”ening in orthopedic care: a concise report from a 300-bedded tertiary care teaching center. Eur J Orthop Surg Traumatol.

[9] Drake TM, Claireaux HA, Khatri C, Chapman SJ (2016). WhatsApp with patient data transmitted via instant messaging?. Am J Surg.

[10] Kunde L, McMeniman E, Parker M (2013). Clinical photography in dermatology: ethical and medico-legal considerations in the age of digital and smartphone technology. Australas J Dermatol.

[11] (2015). Medical Board of Australia.

[12] (2014). Australian Medical Association.

