# Supplementary material for: The Use of Communication Apps by Medical Staff in the Australian Health Care System: Survey Study on Prevalence and Use
Source: JMIR Med Inform. 2018 Feb 9;6(1):e9. doi: 10.2196/medinform.9526 (PMC5889814; doi:10.2196/medinform.9526)
Supplement: Multimedia Appendix 1 [file medinform_v6i1e9_app1.pdf]

Clinical Use of Smartphone Communication Applications

Demographics

1. What level of training are you at?

- ◯ Medical student
- ◯ Intern
- ◯ Resident
- ◯ Registrar
- ◯ Fellow
- ◯ Consultant

Clinical Use of Smartphone Communication Applications

Demographics

2. What area are you working in currently?

- ◯ Medicine
- ◯ Surgery
- ◯ Paediatrics
- ◯ O&G
- ◯ Rehab
- ◯ Radiology
- ◯ Anaesthesiology
- ◯ Other (please specify)

Clinical Use of Smartphone Communication Applications

Demographics

3. What is your main area of practice?

- ◯ Medicine
- ◯ Surgery
- ◯ Paediatrics
- ◯ O&G
- ◯ Rehab
- ◯ Radiology
- ◯ Anaesthesiology
- ◯ Other (please specify)

Clinical Use of Smartphone Communication Applications

Demographics

Clinical Use of Smartphone Communication Applications

Demographics

4. What sort of smartphone do you own?

- ◯ iPhone
- ◯ Android
- ◯ Blackberry
- ◯ Other (please specify)

Clinical Use of Smartphone Communication Applications

Communication Apps

5. Do you USE any of the following communication applications (apps) for CLINICAL / WORK purposes?

- ◯ WhatsApp
- ◯ Viber
- ◯ MedX
- ◯ Other (please specify)
- ◯ Slack
- ◯ No, I don't have or use any communication apps on my smartphone

Clinical Use of Smartphone Communication Applications

Communication Apps

Clinical Use of Smartphone Communication Applications

Communication Apps

6. What is the MAIN communication app you use to communicate for CLINICAL purposes?

- ◯ WhatsApp
- ◯ Viber
- ◯ MedX
- ◯ Slack
- ◯ Other (please specify)

Clinical Use of Smartphone Communication Applications

Frequency of Use of Communication Apps for Clinical Purposes

7. How frequently do you use a communication app for CLINICAL purposes?

- daily
- weekly
- monthly
- yearly
- Only if someone messages me first

Clinical Use of Smartphone Communication Applications

Frequency of Use of Communication Apps for Clinical Purposes

8. How many messages on average would you send per day?

- One to ten
- Ten to 20
- 20-30
- 30-40
- 40-50
- >50

Clinical Use of Smartphone Communication Applications

Frequency of Use of Communication Apps for Clinical Purposes

9. How many messages on average would you send per week?

- One - ten
- Ten - 20
- 20-30
- 30-40
- 40-50
- >50

Clinical Use of Smartphone Communication Applications

Frequency of Use of Communication Apps for Clinical Purposes

10. How many messages on average would you send per month?

- One - ten
- Ten - 20
- 20-30
- 30-40
- 40-50
- >50

Clinical Use of Smartphone Communication Applications

Frequency of Use of Communication Apps for Clinical Purposes

11. How many messages on average would you send per year?

- ◯ One - ten
- ◯ Ten - 20
- ◯ 20-30
- ◯ 30-40
- ◯ 40-50
- ◯ >50

Clinical Use of Smartphone Communication Applications

Clinical Use of Communication Apps

12. Do you use communication apps in CLINICAL situations to communicate with colleagues about the MANAGEMENT of patients?

- ◯ yes
- ◯ no

13. Do you use communication apps in CLINICAL situations to update colleagues about patient RESULTS?

- ◯ yes
- ◯ no

14. Do you use communication apps in CLINICAL situations to facilitate clinical HANDOVER?

- ◯ yes
- ◯ no

15. Do you belong to any ‘groups’ within your communication app that primarily relate to a work team? (Such as a ‘colorectal specialty’ group, or ‘respiratory team’ group)

- ◯ yes
- ◯ no

Clinical Use of Smartphone Communication Applications

Clinical Use of Communication Apps

16. Do you use these 'groups' to keep the team updated on patient care?

- yes
- no

17. What types of clinical information do you ever put on communication apps for CLINICAL purposes (You can chose more than 1 response)

- Images of patient stickers
- Patient names
- Patient UR numbers
- Pictures (work related)
- Admission notes
- Imaging reports
- Pictures of radiological images
- Reports of other investigations (echocardiography, endoscopy etc)
- ECGs
- Pathology reports
- Microbiology reports
- Questions to colleagues about management
- Operation reports
- Answers to colleagues about management
- Other (please specify)

Clinical Use of Smartphone Communication Applications

Attitudes Towards Communication Apps

18. I would like to take a photograph of a wound of a patient on my smartphone to send to the plastics registrar.
In this situation:

- Consent is not required
- Verbal consent from the patient is required
- Verbal consent from the patient is required and I must document this in the patient notes
- Verbal and written consent from the patient is required (using a hospital consent form)
- Taking photographs of a patient's wound is not permitted, even with patient consent
- Other (please specify)

19. I would like to take a laparoscopic photograph of a patient's appendix to put in the patient notes. Select the most correct answer:

- ○ No patient consent is required
- ○ Patient consent is required verbally pre-operatively
- ○ Patient consent is required verbally post-operatively (with the photograph being destroyed if they do not give consent)
- ○ Written patient consent is required using a hospital consent form pre-operatively
- ○ Other (please specify)

20. I think that patient information that is communicated on a communication app is:

21. Have you ever felt concerned that you may get ‘into trouble’ for using a communication application for clinical purposes?

- ○ yes
- ○ no

22. Do you see any downsides or negatives to using communication apps on your smartphone for CLINICAL purposes?

- ○ yes
- ○ no

Clinical Use of Smartphone Communication Applications

Attitudes Towards Communication Apps

23. Which of the following do you think are downsides to the use of communication apps in CLINICAL situations

- ☐ Use of internet data on your phone
- ☐ Needing to login to hospital wifi to use the application
- ☐ Needing to login to the application each time you use it (if applicable)
- ☐ Potential risk to patient confidentiality
- ☐ One sided communication (ie no confirmation that the other person has received the message)
- ☐ Missing updates on patient care plans
- ☐ Expectation that if it's been discussed on the communication application that you must be aware (ie may not be reading it on day off)
- ☐ Other (please specify)

24. Do you think there are positives to using communication apps on your smartphone for CLINICAL purposes?

- ○ yes
- ○ no

Clinical Use of Smartphone Communication Applications

Attitudes Towards Communication Apps

25. Which of the following do you think are positives to the use of communication apps in CLINICAL situations

- [ ] Able to communicate quickly with others
- [ ] Less interruptions for yourself/colleagues (can check messages when free rather than phone calls)
- [ ] Portability
- [ ] Facilitate patient handover
- [ ] Keeping the team updated on patient progress
- [ ] Able to update colleagues on patient results easily
- [ ] Able to ask questions of colleagues easily
- [ ] Other (please specify)

26. I am more likely to use a communication application if: (you can select more than one answer)

- [ ] Easy to login
- [ ] Communication ‘groups’ can be created
- [ ] Able to see who has read your question/comment
- [ ] Free application
- [ ] The majority of my colleagues are already using the app
- [ ] Ability to ‘mute’ conversations for different periods of time (ie when not at work)
- [ ] I can share pictures / images on the app easily
- [ ] It complies with the hospital privacy policies
- [ ] Other (please specify)

27. Did you know that patient data that is stored on an overseas server (such as Facebook, WhatsApp etc) is against Australia's privacy regulations?

- ( ) Nope, no idea
- ( ) Yes

Clinical Use of Smartphone Communication Applications

MedX

28. Are you aware of the communication app 'MedX'?

- yes
- no

Clinical Use of Smartphone Communication Applications

MedX

29. Do you use MedX as your MAIN form of communication for CLINICAL purposes?

- yes
- no

Clinical Use of Smartphone Communication Applications

MedX

30. Why do you use MedX over other communication apps for CLINICAL purposes?

31. Why do you use other communication apps over MedX for CLINICAL purposes?

32. Are you aware of any hospital policies that exist regarding the use of communication apps for CLINICAL communication:

- yes
- no

Clinical Use of Smartphone Communication Applications

Communication Apps

33. Why DON'T you use communication apps for CLINICAL purposes?

- ◯ Concerns about patient safety
- ◯ Colleagues not using communication apps
- ◯ Other methods of communication used (e.g face to face meetings, text, email, paging)
- ◯ I don't want to use / do not have internet/ data on my smartphone
- ◯ Other (please specify)
